# Mosquito Traps: An Innovative, Environmentally Friendly Technique to Control Mosquitoes

**DOI:** 10.3390/ijerph14030313

**Published:** 2017-03-18

**Authors:** Brigitte Poulin, Gaëtan Lefebvre, Camille Muranyi-Kovacs, Samuel Hilaire

**Affiliations:** 1 Tour du Valat Research Institute for the conservation of Mediterranean wetlands, Le Sambuc, 13200 Arles, France; lefebvre@tourduvalat.org (G.L.); muranyi.camille@gmail.com (C.M.-K.); hilaire@tourduvalat.org (S.H.); 2 Université Montpellier, Faculté des Sciences, Place Eugène Bataillon–CC437, 34095 Montpellier cedex 5, France

**Keywords:** Bti-spraying alternative, Camargue, environmental impacts, mosquito control, Techno Bam traps

## Abstract

We tested the use of mosquito traps as an alternative to spraying insecticide in Camargue (France) following the significant impacts observed on the non-target fauna through Bti persistence and trophic perturbations. In a village of 600 inhabitants, 16 Techno Bam traps emitting CO_2_ and using octenol lures were set from April to November 2016. Trap performance was estimated at 70% overall based on mosquitoes landing on human bait in areas with and without traps. The reduction of *Ochlerotatus caspius* and *Oc. detritus*, the two species targeted by Bti spraying, was, respectively, 74% and 98%. Traps were less efficient against *Anopheles hyrcanus* (46%), which was more attracted by lactic acid than octenol lures based on previous tests. Nearly 300,000 mosquitoes from nine species were captured, with large variations among traps, emphasizing that trap performance is also influenced by surrounding factors. Environmental impact, based on the proportion of non-target insects captured, was mostly limited to small chironomids attracted by street lights. The breeding success of a house martin colony was not significantly affected by trap use, in contrast to Bti spraying. Our experiment confirms that the deployment of mosquito traps can offer a cost-effective alternative to Bti spraying for protecting local populations from mosquito nuisance in sensitive natural areas.

## 1. Introduction

*Bacillus thuringiensis israelensis* (Bti) is the most selective and least toxic larvicide currently available to control mosquitoes [1]. However, its sustained use in wetland-dominated areas has revealed strong indirect impacts on animal species that depend on small dipterans and/or their predators for breeding and survival [2]. In Camargue (Rhône delta, southern France), the spraying of 2500 out of 25,000 ha of mosquito larval biotopes with Bti has led to a significant 30%—60% decrease in the breeding success of house martins [3], in the richness and abundance of odonates [4], as well as the invertebrate prey available to reed passerines [5]. Mosquito control in Camargue was initiated 50 years after its implementation on the French Mediterranean coast, on the assumption that Bti use would permit, in contrast to chemical insecticides, a reconciliation of nature protection with human comfort. However, the observed impact on the non-target fauna due both to the mosquito reduction and the collateral effects on benthic chironomids following Bti persistence in the sediments [6,7] is calling for alternative solutions. 

Various mosquito traps are commercially available for public consumers to reduce the mosquito nuisance and/or decrease the risk of mosquito-borne illness [8]. Could a network of traps be used as a protecting belt around inhabited areas to improve human comfort while preserving wetland biodiversity? Deploying mosquito traps in urban areas appeared as a cost-efficient alternative to traditional mosquito control in Camargue, where small villages and towns are typically surrounded by thousands of hectares of wetlands potentially producing mosquitoes. A first prototype adapted to collective use and inspired from the functioning of traps available for individual consumers was developed and patented by Techno Bam (http://techno-bam.net/fr/), a small local business, in 2014. After some initial tests in 2015, 16 of these traps were deployed in a hamlet of 600 inhabitants and operated during the whole mosquito season in 2016. This study reports on the efficacy and environmental impact of this experiment as an innovative way to control mosquitoes in an area reputed for its high mosquito density during several months of the year. 

## 2. Materials and Methods

**Figure 1:**
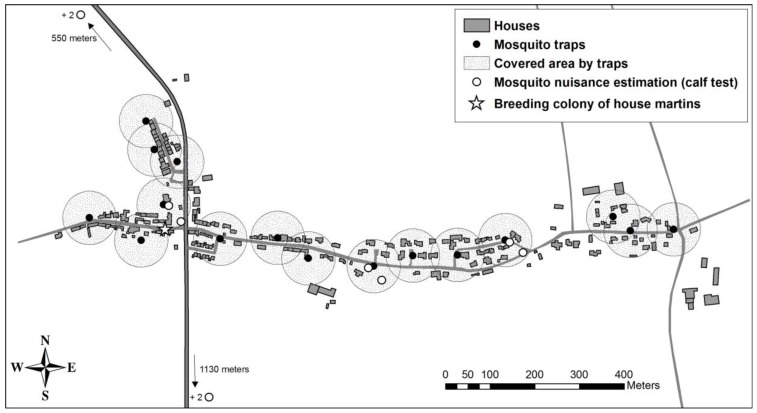
Deployment of the 16 Techno Bam traps in the Sambuc hamlet in 2016 with their 60-m attraction radius for mosquitoes relative to location of human bait tests and the breeding colony of house martins (*Delichon urbicum*).

Mosquito traps: Techno Bam traps use octenol-based lures in the form of absorbent beads and release of recycled carbon dioxide from CO_2_ cylinder to attract female mosquitoes. A power-supplied impellor fan sucks the female mosquito into a net of 1 × 0.5 mm mesh. Because the traps are made for public use, all the material needed for their functioning is concealed into a weather-resistant box locked and riveted to the ground. A total of 16 traps were deployed, covering most of the Sambuc hamlet based on a 60-m attraction radius for mosquitoes (Figure 1). 

Insect samples: Traps were operated from mid-April through late October 2016, with the nets being emptied from three to five times a week (*n* = 1380 samples). Fresh samples were brought to the laboratory and weighted. Each week, samples from three traps (*n* = 86) were examined under a stereoscope to determine the number of species and individuals of biting dipterans, as well as the number of non-target insects identified to taxonomic order. From these samples we calculated a mean bodyweight for mosquitoes (2.55632 mg) that was used to extrapolate their numbers from the weighed samples. 

**Figure 2:**
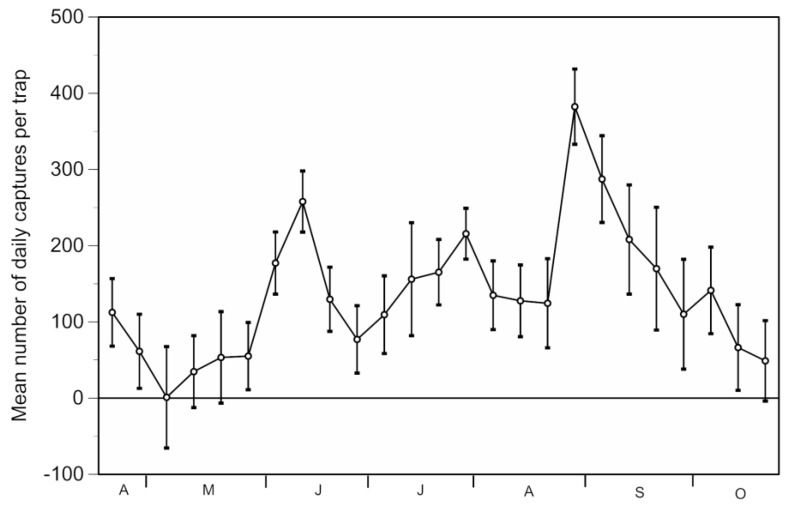
Weekly variation in the mean number of mosquitoes captured daily in each of the 16 Techno Bam traps located at Sambuc from April through October 2016.

Trap performance: While the number of mosquitoes trapped can be a relative measure of trap performance (e.g., for comparing different trap models [9]), the main criteria for assessing absolute performance should be the reduction in biting pressure on humans. Accordingly, trap performance was assessed by comparing the number of mosquitos landing on human baits (calf test) during a 10 min period at three locations in the hamlet (at 10 m and 40 m from trap position) and two locations outside the hamlet (at 550 m and 1130 m from the nearest trap position) before (2015) and during (2016) the experiment (Figure 2). We collected 60 samples in 2015 before trap installation, and 334 samples during the 2016 experiment. Sampling was done at least once a week, should environmental conditions be favorable (low wind, no rain, presence of mosquitoes outside twilight activity peak). Calf tests were made simultaneously by one or several observers, with the same observer(s) covering systematically control and treated points in an alternate manner during each sampling period. All mosquitoes landing on human baits were collected with a mouth aspirator, counted and identified to species. Trap performance was assessed globally and for each mosquito species by estimating the percent decrease in the mean number of biting attempts at treated relative to control areas using Generalized Linear Models with a nested ANOVA design (Statistica V12, Stat Soft Inc. Maisons-Alfort, France), where sites and dates were nested in treatment (fixed factor).

Environmental impacts: We estimated direct effects of Techno Bam traps based on the presence of non-target insects captured in the traps, as well as indirect effects based on the breeding success of a colony of house martins *(Delichon urbicum)* nesting in the treated area (Figure 1). Breeding success was estimated by visiting 21 nests twice a week from 12 May to 27 August to determine the number of fledged young from all breeding attempts in the season. Mean number of young produced by nest was compared to the breeding success observed at the same site prior to the trap experimentation in 2015, as well as to the breeding success observed at two control sites (including the Sambuc colony) and two sites surrounded by Bti-sprayed wetlands that were monitored from 2009 to 2011^3^. These analyses were made using a GLM with a nested ANOVA design where site and year were nested in treatment (fixed factor).

## 3. Results

### 3.1. Trap Performance

The estimated number of mosquitoes trapped daily varied over time, with three peaks observed in June, July and August (Figure 2). Overall, an estimated number of 299,408 mosquitoes was captured, with mean a daily capture rate per trap ranging from one mosquito in early May to 382 mosquitoes in late August.

Mean capture rates also varied spatially among traps, ranging from 24 to 399, depending upon their location in the hamlet. The highest number of mosquitoes caught in a single day in one trap was 4300 in late August. 

Prior to trap installation in 2015, the relative mosquito nuisance was higher at Sambuc (mean 8.6 ± 1.3 SE) than at the control sites (mean 4.1 ± 1.5 SE) located 550 and 1130 m from the hamlet (F _(1,32)_ = 5.21; *p* = 0.029). After trap installation, however, the relative mosquito nuisance was significantly lower at Sambuc compared to the control sites (F _(1,294)_ = 18.46; *p* < 0.0001). Overall, the mosquito nuisance was reduced by 70%, with a mean of 4.1 biting attempts/10 min at 10–40 m from the traps, compared to 14.1 at control sites. Calf tests provided similar results when conducted at 10 m and 40 m from the traps (F _(1,110)_ = 0.252, *p* = 0.62), hence these data were combined in the analyses.

**Figure 3:**
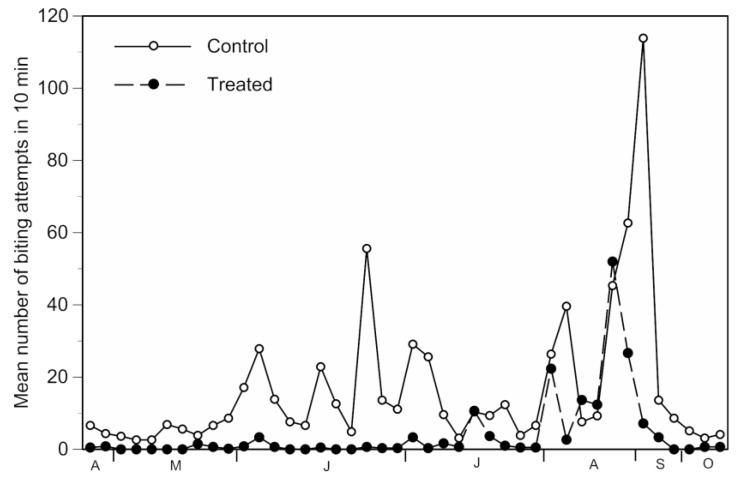
Temporal variation in the mean number of biting attempts at treated (10–40 m from traps) and control (550–1130 m from traps) sites from April to October 2016.

On a weekly basis, the mosquito nuisance was kept at very low levels until mid-July in the area covered by traps (Figure 3), despite various peaks in mosquito nuisance obtained at the control sites (calf tests) and confirmed at Sambuc through mosquito captures in the traps (Figure 2). However, three peaks of mosquito nuisance with over 10 biting attempts/10 min were observed in July, and early and late August at Sambuc (Figure 3). In these cases, trap use permitted us to reduce the level and duration of the mosquito nuisance but not to eliminate it completely.

Nine mosquito species were captured in traps and on human bait (Table 1). All species present in both the control and treated areas showed a reduced abundance in treated areas, the latter being highly significant for four species. The species mainly responsible for the mosquito nuisance were well controlled by the use of traps, their reduction rate varying from 74% to 98% with the exception of Anopheles hyrcanus, which was responsible for the peak observed near the end of the mosquito season (Table 1). Trap performance was also lower for Culex spp., especially Cx. Modestus, which accounted for 0.14% of captures in traps and 4.16% of captures on human bait. Finally, although there were a few tiger mosquitoes, Aedes albopictus, in the hamlet (two individuals captured in traps and on human bait), the absence of this urban species at control sites makes the calculation of a reduction rate relative to trap use impossible.

### 3.2. Environmental Impacts of Techno Bam Traps

#### 3.2.1. Direct Effects

**Figure 4:**
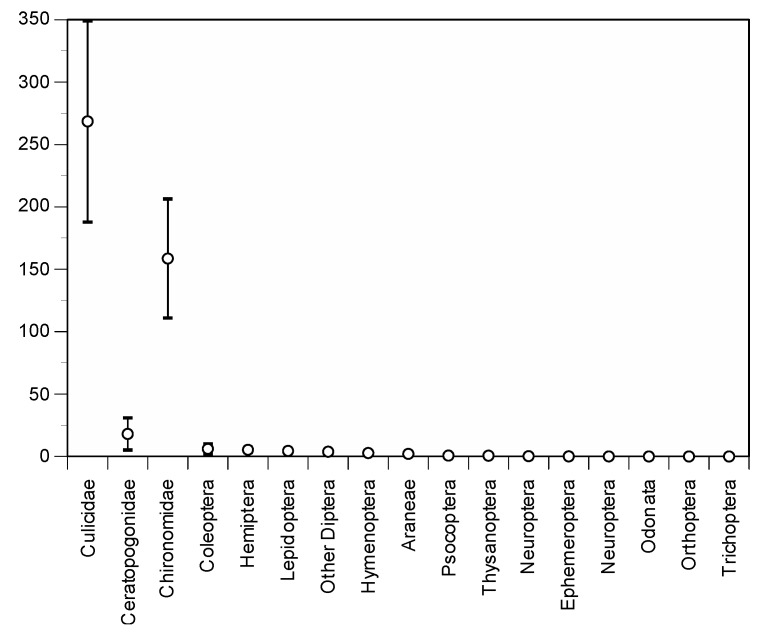
Mean daily captures from each taxonomic group based on 39,941 items identified in 86 Techno Bam trap samples at the Sambuc in 2016.

We counted and identified 39,941 insects in the 86 trap samples that were examined in detail. Of these, 23,098 (57.8%) were mosquitoes, 1499 (3.8%) were Ceratopogonidae and 15,359 (38.4%) were non-target insects (Figure 4). Non-target insects were dominated (85.7%) by non-biting, small Chironomidae, which were occasionally captured by the hundreds, especially in one of the traps located under a street light. Their capture was detected only after adding a second net of a smaller mesh size (1 × 0.5 mm instead of 1.5 × 1 mm) to avoid Ceratopogonidae from escaping from the traps. Fourteen other taxa were also captured in roughly equal proportions, representing globally 5.5% of all the captures in the traps (Figure 4).

#### 3.2.2. Indirect Effects

**Figure 5:**
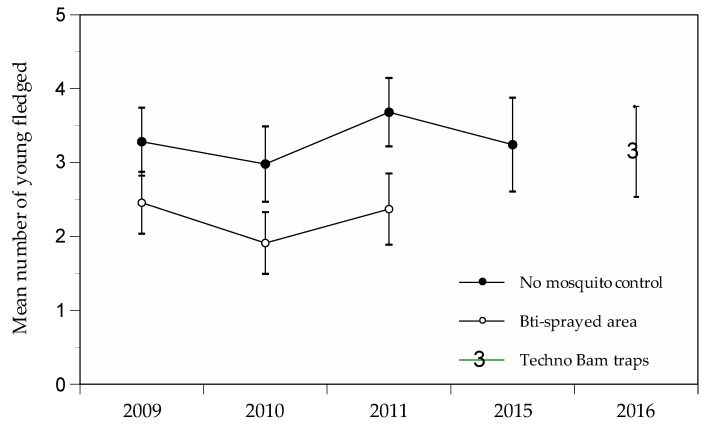
Mean breeding success of house martin in two Bti-sprayed areas and two control areas (including Sambuc) in Camargue between 2009 and 2015 and with Techno Bam traps in 2016.

The house martin *Delichon urbicum* is a migratory aerial insectivore that breeds colonially in human-inhabited areas. It feeds upon various arthropod species that are caught on the wing within 500 m from the nest [10,11]. In Camargue, breeding extends from early May (laying period) to mid-August (fledging of young from second breeding attempt), with a third of the chick diet being composed of small Nematocera [3]. While mosquito control using Bti spraying had a significant impact on the breeding success of house martins (*F_(2, 212)_* = 16.2, *p* < 0.0001), the use of traps revealed a similar breeding success to the one reported outside the Bti-sprayed area (Figure 5). The mean number of young fledged per nest was 3.3 at sites without mosquito control, 3.1 at the site with Techno Bam traps and 2.2 at sites treated with Bti. According to post-hoc Fisher’s LSD tests, breeding success at Sambuc in 2016 (with traps) was not different (*p* = 0.24) from that observed in the preceding years at the control sites (including Sambuc), but differed significantly (*p* = 0.03) from that of sites surrounded by Bti-sprayed wetlands. 

## 4. Discussion

Although mosquito traps using CO_2_ and olfactive lures to attract mosquitoes are commonly used in surveillance programs [9,12,13], few studies have experimentally tested their usefulness as a means of mosquito control on a relatively large spatial scale [14]. Considering the high environmental and economic costs of spraying insecticide, this technique appears as the most promising, with a performance similar to traditional methods for controlling mosquitoes [15]. 

The use of 16 Techno Bam traps spread over 1.5 km within a hamlet of 600 inhabitants allowed us to reduce the mosquito nuisance by 70%. This performance, assessed by comparing the number of mosquitoes landing on human bait within and outside the hamlet, before and during trapping operations, was associated with the catch of nearly 300,000 females from nine mosquito species. 

Mosquito peaks were nevertheless observed over the six-month sampling season in the controlled areas. These were mostly related to *Anopheles hyrcanus,* which accounted for 81% of the residual nuisance observed in late August. The lower trap performance against this species (46% reduction) could be related to the type of olfactive lure used [16]. When *Anopheles hyrcanus* is excluded from our calf-test samples, the performance of the Techno Bam traps reaches 85% in terms of nuisance reduction. An unpublished experiment comparing the performance of the first Techno Bam prototype with Biogent Sentinel traps suggested that lures using lactic acid are more effective against *An. hyrcanus* than those using octenol. 

The large discrepancy in the mean number of daily captures among traps (range 24–399) suggests that the performance is influenced by trap placement within the hamlet. Sunlight has been shown to negatively influence the capture probability of *Aedes albopictus* [17], but literature on this subject is relatively scant. We would also expect wind exposure and the presence of vegetation to influence the mosquito capture rates. 

Testing this new approach to mosquito control in Camargue was motivated by the significant impacts revealed by Bti spraying on natural predators of mosquitoes and chironomids [2,3,4,5]. In contrast to larvicide spraying of natural areas, the environmental impact of traps is expected to be negligible, being mostly limited to the impoverished fauna found in urbanized areas where the traps are located. Some 86% of the non-target insects captured in the traps were very small chironomids attracted by street lights. Because non-target insects are presumably not attracted by carbon dioxide, only those individuals flying incidentally close to the trap will be caught by the fan aspiration. Hence, although small chironomids accounted for a third of all captures, the proportion caught was presumably negligible relative to their local abundance. Techno Bam traps did not affect the breeding success of house martins nesting colonially at the proximity of traps. These results suggest that the local use of traps has no impact on insects fed to nestlings in contrast to the Bti spraying of wetlands surrounding urban areas where these birds are nesting [3].

## 5. Conclusions

This study provides the first experimental data on the performance of a public network of mosquito traps as a means of mosquito control to improve human comfort in a locality. The lack of data on the efficacy of Bti spraying, which has been carried out since 2006 in Camargue, does not allow us to quantitatively compare the performance of both techniques. However, traps are qualitatively more versatile as they capture all mosquito species potentially causing a nuisance in human-inhabited areas (Bti spraying targets only *Ochlerotatus caspius* and *Oc. Detritus),* they are more economical in spite of their relatively high maintenance costs, and they have a negligible impact on wildlife. Because they are located in human-inhabited areas, mosquito traps could provide a useful complementary tool for the control of container-inhabiting species such as *Aedes albopictus* and *Aedes aegypti,* which pose public health problems, and for which traditional integrated mosquito management approaches based on larvae control are inefficient [18,19,20]. Our experiment suggest that the observed 70% reduction in the mosquito nuisance could be increased by combining different olfactive lures, by optimizing the position of traps relative to environmental conditions, and by increasing trap numbers to improve the protecting belt effect. 

## Figures and Tables

**Table 1:** Capture rates and trap performance for the different mosquito species sampled in 2016.

	Traps	Human bait (calf tests)					
	%	% biting	Mean (SE) biting rate/10 min		Reduction	ANOVA Statistics	
Mosquito species	captures	attempts	Control	Treated	Rate (%)	*F_(1,294)_*	*p* value
*Ochlerotatus caspius*	82.76	51.39	7.68 (0.92)	1.97 (0.54)	74	28.7	<0.00001
*Anopheles hyrcanus*	8.73	35.27	3.44 (1.54)	1.87 (0.89)	46	0.4	0.37
*Aedes vexans*	4.76	2.05	0.57 (0.07)	0.03 (0.04)	94	38.7	<0.00001
*Culex pipiens*	1.99	0.23	0.03 (0.01)	0.01 (0.01)	67	1.57	0.21
*Oc. detritus/coluzzii*	1.40	6.77	1.86 (0.19)	0.03 (0.11)	98	70.8	<0.00001
*Culex modestus*	0.14	4.16	0.44 (0.41)	0.22 (0.24)	50	0.22	0.64
*Culiseta annulata*	0.06	0.03	0.017 (0.01)	0	100	6.05	0.014
*Aedes albopictus*	0.01	0.07	0	0.007 (0.01)		0.5	0.48
*Anopheles maculipennis*	0.14						
*Coquillettidia richiardii*		0.03	0.017 (0.01)	0	100	6.06	0.014
Total	299,408	3051	14.06 (1.16)	4.15 (1.99)	70.5%	18.46	0.00002
